# Traumatic Aneurism, Clinical Remarks by C. C. F. Gay, M. D.

**Published:** 1879-12

**Authors:** Fred. Peterson

**Affiliations:** House Surgeon


### CLINICAL REPORTS.

---

### TRAUMATIC ANEURISM.

LIGATION OF THE FEMORAL ARTERY—GANGRENE—AMPUTATION—HOT WATER TREATMENT IN TRAUMATIC GANGRENE—CLINICAL REMARKS BY C. C. F. GAY, M. D., SURGEON TO THE BUFFALO GENERAL HOSPITAL.

REPORTED BY FRED. PETERSON, M. D., HOUSE SURGEON.

THOMAS GRABNER entered the hospital January 17th, 1879; aged 23; American; married; butcher.

While at work with an ordinary butcher-knife, the instrument accidentally slipped, making a penetrating wound in the inferior portion of scarpa's triangle and injuring the larger femoral vessels in the vicinity. This had occurred eighteen days before admittance to the ward. A false aneurism had formed, and after a consultation, Dr. J. F. Miner cut down upon it. The incision was made from Poupart's ligament, eleven inches in length toward the inner tuberosity of the femur. About a pint of coagula was removed. In three days secondary hemorrhage took place and the femoral artery was ligated close upon, or a little beneath, Poupart's ligament. The leg seemed to improve for several weeks and the long incision healed rapidly, while the collateral circulation had every appearance of being established.

March 1. Service of Dr. Gay. The great toe had sloughed to such a degree that amputation was necessary, and the lesser toes gradually assumed the same gangrenous condition. Hot water irrigation was recommended by Dr. Gay and a continuous stream, at a temperature of 130° to 140° Fahr., played upon it night and day until the 26th of March, manifestly *delaying* the fatal progress. March 23d, two other toes were pulled off. Ultimately, however, the heel and dorsal surface of the foot lost vitality, more it seemed from innervation than from paucity of blood, for the circulation was good and the temperature of the leg and foot always from 1° to 2° higher than that of the healthy extremity. A consultation of the hospital staff, March 20th, decided in favor of amputation, but considered it proper to delay the operation for a little time to see if there might be any improvement.

March 27. Dr. Gay amputated in the presence of the Staff at the junction of the upper with the middle third, making a good, healthy stump. Three days afterward the bandage was removed, and the posterior flap found gangrenous but with a line of demarkation within an inch of the margin. His pulse was too frequent to be counted. His temperature varied from 103° to 105° Fahr. Stimulants and nutritious diet were ordered. Morphine gr. ½ every two hours was taken for a few days. The stump was immersed in water at a temperature of 100° to 110° Fahr.

April 5. The ligature about the femoral was removed, and with difficulty, after having remained there nine weeks.

April 13. The stump was removed from the water and poulticed with flaxseed until the 16th inst., when healthy granulations were visible. Was given ferri et quinias citras gr.v=gr.v three times daily. Simple cerate dressing was now recommended to be used.

May 3. Tried grafting, putting in seven points of skin taken from the arm; it was a failure. Two small pieces of bone have come away.

June 12. Patient has grown fat, is able to sit up and has been for many weeks, and now only awaits the tedious process of the skin covering the stump. Grafting was repeated, and the stump covered.

REMARKS OF DR. GAY AT HIS CLINIC.

The case of Grabner possesses much interest. At the time of the first consultation the patient wore a tourniquet loosely applied to the femoral artery; the thigh was distended from infiltration of blood; the penetrating wound was outside the line of the superficial femoral artery; there was pulsation and the characteristic aneurismal thrill. No thrill was heard over the artery of the opposite limb. Thrill has been known to be present, sometimes, upon both sides when there was aneurism only upon one side; and this is a clinical fact of some interest. There could be no reasonable doubt of the presence of either true or false aneurism.

In cases of aneurism, I regard it most desirable to establish collateral circulation, if possible, previous to ligation.

My aneurism compressor is so constructed that elastic pressure can be applied, and the circulation wholly or partially shut off in the artery to which the instrument is adjusted without ligating the limb.

An interesting clinical fact as any other, probably, in connection with this case, is that of the efficacy of the hot water treatment in traumatic gangrene.

Upon the subject of the employment of hot water in surgery, Dr. Hamilton has reported many cases, some of which I regard as not entirely test cases, and would have ended in recovery undoubtedly without the use of hot water. But in the case of Grabner we present a test case, and there can be no doubt about the character of the disease, nor of the efficacy of the remedy in saving the patient's limb and life, so that we may depart from our usual custom and say: here is a patient "cured."

After complete immersion of the leg for a few days in hot water, pretty uniformly maintained at a temperature of 107° to 110° Fahr., the progress of the gangrene was arrested.

Fomentations and irrigation, as employed in this case, previous to amputation, are not the most effective ways of using hot water as a remedial agent. Irrigation was effected by placing a pail of hot water two feet above the limb, through the bottom of which was a hole for the passage of a rope of wicking, which was to convey the water to the limb. Of course, the temperature of the water was considerably lowered during its transit from the pail to the limb.

After gangrene had made some inroads upon the amputated limb, not only the stump, but a portion of the thigh also, was submerged in a tin trough of hot water. The efficacy of the hot water treatment is, probably, dependent upon complete and continuous submersion, and to this method is attributed the final arrest of the sloughing.

